# Intrinsic Flat and Gromov-Hausdorff Convergence of Manifolds with Ricci Curvature Bounded Below

**DOI:** 10.1007/s12220-016-9742-7

**Published:** 2016-09-28

**Authors:** Rostislav Matveev, Jacobus W. Portegies

**Affiliations:** Max Planck Institute for Mathematics in the Sciences, Leipzig, Germany

**Keywords:** Intrinsic flat distance, Gromov-Hausdorff distance, Ricci curvature, 53C23, 49Q15

## Abstract

We show that for a noncollapsing sequence of closed, connected, oriented Riemannian manifolds with Ricci curvature bounded below and diameter bounded above, Gromov-Hausdorff convergence agrees with intrinsic flat convergence. In particular, the limiting current is essentially unique, has multiplicity one, and mass equal to the Hausdorff measure. Moreover, the limit spaces satisfy a constancy theorem.

In this manuscript, we consider the class ${\mathcal {M}}(n, \Lambda , v, D)$ of *n*-dimensional, closed, connected, oriented Riemannian manifolds *M* with

$$\begin{aligned} {{\mathrm{Ric}}}_{M} \ge -(n-1) \Lambda , \quad {{\mathcal {H}}}^n(M) \ge v > 0, \quad {\text {Diam}}(M) \le D, \end{aligned}$$

and show that for a sequence of manifolds in this class, Gromov-Hausdorff convergence essentially agrees with intrinsic flat convergence.

The intrinsic flat distance was introduced by Sormani and Wenger [13], and relates to the flat distance as the Gromov-Hausdorff distance relates to the Hausdorff distance.

In general, there are significant differences between Gromov-Hausdorff convergence and intrinsic flat convergence: the intrinsic flat limit may be noncompact, there are sequences of Riemannian manifolds that do have an intrinsic flat limit but do not have a Gromov-Hausdorff limit, the intrinsic flat limit is always rectifiable, etc. However, in the presence of a uniform lower bound on the Ricci curvature and the volume, the theory on the structure of Gromov-Hausdorff limits developed by Cheeger and Colding [3–5] suggests that the two concepts may not differ all that much.

The first result in this direction goes back to Sormani and Wenger [12]. They show that if a sequence of manifolds $M_i \in {\mathcal {M}}(n, \Lambda = 0, v, D)$ with *nonnegative* Ricci curvature converges in the Gromov-Hausdorff distance to a metric space *X*, then a subsequence will converge in the intrinsic flat distance to an integral current space $(X, d_X, T)$: a metric space *X* endowed with an integral current *T* (in the sense of Ambrosio-Kirchheim [1]), that is completely settled (meaning *X* is exactly the set of positive *n*-dimensional lower density for *T*). Recently, Munn [8] has obtained a similar result for sequences of manifolds with a uniform, two-sided bound on the Ricci curvature.

Li and Perales [7] have proved that for a sequence of integral current spaces for which the metric spaces are Alexandrov spaces of nonnegative curvature and have a uniform diameter upper bound, either the sequence converges in the intrinsic flat distance to the zero space, or a subsequence converges in both the Gromov-Hausdorff and the intrinsic flat distance, and the underlying metric spaces in the limit are the same.

This manuscript extends the results by Sormani and Wenger [12] and Munn [8] to sequences of manifolds with an arbitrary uniform lower bound on the Ricci curvature and additionally shows that the limiting current is essentially unique, has multiplicity one, and has mass equal to the Hausdorff measure on the limiting space.

Before we state our main results more precisely, we need to introduce some notation. The class ${\mathcal {M}}(n, \Lambda , v, D)$ is precompact both in the Gromov-Hausdorff distance and in the intrinsic flat distance [12]. We denote the completions with respect to these distances respectively by ${\mathcal {M}}^{GH}(n, \Lambda , v, D)$ and ${\mathcal {M}}^{IF}(n, \Lambda , v, D)$.

There is an involution $\iota$ acting on the space ${\mathcal {M}}^{IF}(n, \Lambda , v, D)$ by reverting the orientation of the current. We denote by ${\mathcal {M}}^{IF/\iota }(n, \Lambda , v, D)$ the quotient metric space obtained from ${\mathcal {M}}^{IF}(n, \Lambda , v, D)$ by identifying every integral current space with its image under the involution.

Our first main theorem is the following.

## Theorem A

The map $F : {\mathcal {M}}^{IF/\iota } (n, \Lambda , v, D) \rightarrow {\mathcal {M}}^{GH}(n, \Lambda , v, D)$ given by

$$\begin{aligned} (X, d_X, T)\mapsto X \end{aligned}$$

is well-defined and is a homeomorphism.

Additionally, for all $(X, d_X, T) \in {\mathcal {M}}^{IF}(n, \Lambda , v, D)$,

(i) the set of positive lower-density $\mathrm{{set}}(T)$ of *T* equals *X*,
(ii) the mass measure $\Vert T\Vert$ equals the Hausdorff measure ${{\mathcal {H}}}^n$ on *X*,
(iii) the multiplicity of *T* is equal to 1, ${{\mathcal {H}}}^n$-a.e.

This theorem implies the aforementioned results by Sormani and Wenger and Munn. Yet it also illustrates that the currents are just going along for the ride: up to the involution, the currents are uniquely determined by the underlying metric spaces.

From the fact that the mass measure $\Vert T\Vert$ equals the Hausdorff measure ${{\mathcal {H}}}^n$, it follows that it is the limit (in a weak sense) of the Riemannian volume measures of the manifolds in the approximating sequence. Indeed, Cheeger and Colding [3, Theorem 5.9] have shown that under Gromov-Hausdorff convergence, the Riemannian volume measures on the manifolds converge to the Hausdorff measure on the limit space.

We believe that the idea of the original proof by Sormani and Wenger in [12] can be used to extend their result to the case of Ricci curvature bounded below by an arbitrary constant. The important ingredients in their proof, such as the application of a volume estimate by Colding [6, Corollary 2.19] and Perelman’s Main Lemma in [9], are applicable to manifolds of almost nonnegative curvature, and therefore they can be applied after scaling. To our knowledge, this was so far unknown. Our proof will differ from the one by Sormani and Wenger and does not use Perelman’s Main Lemma.

Our techniques also allow to prove the uniqueness of the limiting current and the following local constancy theorem.

## Theorem B

Let $(X, d_X, T) \in {\mathcal {M}}^{IF}(n, \Lambda , v, D)$. For all $q \in X$, and every integral current *S* on *X* such that $\Vert \partial S \Vert (B_t(q)) = 0$ for some $t > 0$, there exists an integer $k \in {\mathbb {Z}}$ such that $S = k T$ on $B_t(q)$.

Alternatively, Theorem B may be interpreted as stating that the local top-dimensional homology of the space is isomorphic to ${\mathbb {Z}}$.

The structure of the manuscript is as follows. Section 1 gives a coarse background on integral currents in the sense of Ambrosio and Kirchheim [1], integral current spaces and intrinsic flat converge as introduced by Sormani and Wenger [13] and some of the elements we need from the theory on the structure of spaces with Ricci curvature bounded below by Cheeger and Colding [3, 4]. In our notation, we generally try to stick to the notation in these articles.

Theorems A and B are direct consequences of Theorem 4.1 in the text. A crucial ingredient is a link between zero-dimensional slices and the degree, which we will explain in Sect. 2. Earlier work by Sormani and the second author [10] showed that integrals of the flat distance between lower-dimensional slices of currents can be controlled by the flat distances between the original currents. This will imply that the $L^1$-distance between their degrees can be controlled locally. In Sect. 3, we show that Colding’s volume estimate easily translates into an estimate on the degree for manifolds $M \in {\mathcal {M}}(n, \Lambda , v, D)$. In Sect. 4 we combine these two ingredients to give a proof of the main theorem.

## Background

In this section, we review integral currents on metric spaces as introduced by Ambrosio and Kirchheim [1], the intrinsic flat distance introduced by Sormani and Wenger [13] and some theory on the structure of spaces with Ricci curvature bounded below by Cheeger and Colding [3, 4]. The prime purpose of this review is to fix notation. We adhere closely to the notation used in these articles, and therefore the reader familiar with these works could probably understand the rest of the manuscript without reading this section.

### Currents

Let *X* be a complete metric space. For $n \ge 1$, we define the set ${\mathcal {D}}^n(X)$ of all $(n+1)$-tuples $(f, \pi _1, \dots , \pi _n)$ of Lipschitz functions on *X*, where additionally *f* is required to be bounded. For $n = 0$, we define ${\mathcal {D}}^0(X)$ as the set of bounded Lipschitz functions. It can be helpful to think of an element $(f, \pi _1, \dots , \pi _n) \in {\mathcal {D}}^{n+1}$ as an *n*-form $f d \pi _1 \wedge \dots \wedge d\pi _n$.

An *n*-dimensional metric functional is a function $T: {\mathcal {D}}^n(X) \rightarrow {\mathbb {R}}$ such that the map

1 $$\begin{aligned} (f, \pi _1, \dots , \pi _n) \mapsto T(f, \pi _1, \dots , \pi _n) \end{aligned}$$

is subadditive and positively 1-homogeneous with respect to the functions *f* and $\pi _1, \dots , \pi _n$. We denote the vector space of *n*-dimensional metric functionals on *X* by $MF_n(X)$.

The exterior differential *d* maps ${\mathcal {D}}^n(X)$ into ${\mathcal {D}}^{n+1}(X)$ according to

2 $$\begin{aligned} d(f, \pi _1, \dots , \pi _n) := (1, f, \pi _1, \dots , \pi _n). \end{aligned}$$

For $n \ge 1$, the boundary of $T \in MF_n(X)$ is the $(n-1)$-dimensional metric functional denoted by $\partial T$ defined by

3 $$\begin{aligned} \partial T (\omega ) := T (d \omega ), \qquad \omega \in {\mathcal {D}}^{n-1}(X). \end{aligned}$$

If *Y* is another complete metric space and $\Phi : X \rightarrow Y$ is Lipschitz, we define the pullback operator that maps ${\mathcal {D}}^n(Y)$ to ${\mathcal {D}}^n(X)$ by

4 $$\begin{aligned} \Phi ^\# (f, \pi _1, \dots , \pi _n) = (f \circ \Phi , \pi _1 \circ \Phi , \dots , \pi _k \circ \Phi ). \end{aligned}$$

We define the pushforward $\Phi _\# T \in MF_n(Y)$ of $T \in MF_n(X)$ by

5 $$\begin{aligned} \Phi _\# T (\omega ) := T(\Phi ^\# \omega ), \qquad \omega \in {\mathcal {D}}^n(Y). \end{aligned}$$

For $T \in MF_n(X)$ and $\omega = (g, \tau _1, \dots , \tau _k) \in {\mathcal {D}}^k(X)$, with $k \le n$, we define the restriction $T \llcorner \omega \in MF_{n-k}(X)$ by

6 $$\begin{aligned} T \llcorner \omega (f, \pi _1, \dots , \pi _{n-k}) := T(f g, \tau _1, \dots , \tau _k, \pi _1, \dots , \pi _{n-k}). \end{aligned}$$

We say that $T \in MF_n(X)$ has finite mass if there exists a finite Borel measure $\mu$ on *X* such that for all $(f, \pi _1, \dots ,\pi _n) \in {\mathcal {D}}^n(X)$,

7 $$\begin{aligned} |T(f, \pi _1, \dots , \pi _n)| \le \prod _{i=1}^n {\text {Lip}}(\pi _i) \int _X |f| \, d\mu , \end{aligned}$$

where ${\text {Lip}}(\pi _i)$ denotes the Lipschitz constant of $\pi _i$. Moreover, the minimal measure satisfying this bound is called the mass of *T* and is denoted by $\Vert T\Vert$. When *T* has finite mass, it can be uniquely extended to a function on $(n+1)$-tuples $(f, \pi _1, \dots , \pi _n)$ for which *f* is merely bounded Borel, and $\pi _1, \dots , \pi _n$ are Lipschitz.

An *n*-dimensional current *T* is an *n*-dimensional metric functional with additional properties. From the definition by Ambrosio and Kirchheim [1, Definition 3.1], immediately stronger properties may be derived. We choose to only phrase the stronger properties. The space of *n*-dimensional currents forms a Banach space, with respect to the mass norm ${{\mathbf {M}}}(T) = \Vert T\Vert (X)$. We denote the Banach space by ${{\mathbf {M}}}_n(X)$. Every $T \in {{\mathbf {M}}}_n(X)$ satisfies

(i) *T* is multilinear in $(f, \pi _1, \dots , \pi _n)$, and whenever *f* and $\pi _1$ are both bounded and Lipschitz, it holds that
  $$\begin{aligned} T(f, \pi _1, \dots , \pi _n) + T( \pi _1 , f, \dots , \pi _n) = T( 1, f\pi _1, \dots , \pi _n), \end{aligned}$$
  and
  $$\begin{aligned} T( f , \psi _1( \pi ), \dots , \psi _n(\pi )) = T( f \det \nabla \psi (\pi ) , \pi _1, \dots , \pi _n), \end{aligned}$$
  where $\psi = (\psi _1, \dots , \psi _n) \in [C^1({\mathbb {R}}^n)]^n$ and $\nabla \psi$ is bounded;
(ii) The following continuity property is satisfied
  $$\begin{aligned} \lim _{i \rightarrow \infty } T\left( f^i, \pi _1^i, \dots , \pi _n^i\right) = T\left( f, \pi _1, \dots , \pi _n\right) \end{aligned}$$
  whenever $f^i - f \rightarrow 0$ in $L^1(X, \Vert T\Vert )$ and $\pi _j^i \rightarrow \pi _j$ pointwise in *X* with uniformly bounded Lipschitz constant ${\text {Lip}}(\pi _j^i) \le C$;
(iii) The following locality property holds: $T(f, \pi _1, \dots , \pi _n) = 0$ if $\{ f \ne 0\} = \cup _i B_i$ where $B_i$ are Borel and $\pi _i$ is constant on $B_i$.

We say that a sequence of currents $T_i \in {{\mathbf {M}}}_n(X)$ converges weakly to $T \in {{\mathbf {M}}}_n(X)$ if for all $\omega \in {\mathcal {D}}^n(X)$,

8 $$\begin{aligned} \lim _{i \rightarrow \infty } T_i (\omega ) = T( \omega ). \end{aligned}$$

The mass of open sets is lower-semicontinuous under weak convergence, that is for $O \subset X$ open, and $T_i$ converging weakly to *T*,

9 $$\begin{aligned} \liminf _{i\rightarrow \infty } \Vert T_i\Vert (O) \ge \Vert T\Vert (O). \end{aligned}$$

A very important example of an *n*-dimensional current on the Euclidean space ${\mathbb {R}}^n$ is given by the current induced by a function $g \in L^1({\mathbb {R}}^n)$ which we denote by  and is defined by10We say that a current $T \in {{\mathbf {M}}}_n(X)$ is normal if $\partial T \in {{\mathbf {M}}}_{n-1}(X)$.

A subset $S \subset X$ is called countably ${{\mathcal {H}}}^n$-rectifiable if there are compact sets $K_i \subset {\mathbb {R}}^n$ and Lipschitz functions $f_i: K_i \rightarrow X$ such that

11 $$\begin{aligned} {{\mathcal {H}}}^n\left( S \backslash \cup _{i=1}^\infty f_i(K_i) \right) = 0. \end{aligned}$$

We say $T \in {{\mathbf {M}}}_n(X)$ is rectifiable if $\Vert T\Vert$ is concentrated on a countably ${{\mathcal {H}}}^n$-rectifiable set and vanishes on ${{\mathcal {H}}}^n$-negligible Borel sets. We call *T* integer rectifiable if for all $\phi \in {\text {Lip}}(X, {\mathbb {R}}^n)$ and all open $O \subset X$ it holds that  for some $\theta \in L^1({\mathbb {R}}^n, {\mathbb {Z}})$. Finally, the collection of integral currents will consist of all integer rectifiable currents that are also normal. We will denote this collection by $I_n(X)$ and in this manuscript, we will only deal with integral currents.

We denote by $\omega _n$ the (Lebesgue) volume of the unit ball in ${\mathbb {R}}^n$. For a Borel measure $\mu$ on *X*, we define respectively the *n*-dimensional lower and upper density of $\mu$ in $x\in X$ by

12 $$\begin{aligned} \Theta _{n*} (\mu , x) := \liminf _{r \downarrow 0} \frac{\mu (B_r(x))}{\omega _n r^n}, \qquad \Theta _n^*(\mu , x) := \limsup _{r \downarrow 0} \frac{\mu (B_r(x))}{\omega _n r^n}. \end{aligned}$$

If these values coincide, we call the common value the *n*-dimensional density of $\mu$ in *x* and we denote it by $\Theta _n(\mu ,x)$. We define $\mathrm{{set}}(T) \subset X$ as

13 $$\begin{aligned} \mathrm{{set}}(T) := \left\{ x \in X \, | \, \Theta _{n*}(\Vert T\Vert , x) > 0 \right\} . \end{aligned}$$

For an integer rectifiable current *T*, the mass $\Vert T\Vert$ is always concentrated on $\mathrm{{set}}(T)$ and $\mathrm{{set}}(T)$ is rectifiable.

Integer rectifiable currents allow for a parametric representation. It is a simple consequence of Lusin’s theorem and [1, Theorem 4.5] that if *T* is an *n*-dimensional integer rectifiable current, there exist a sequence of compact sets $K_i$, numbers $\theta _i \in {\mathbb {N}}$ and bi-Lipschitz functions $f_i : K_i \rightarrow E$ such that $f_i(K_i) \cap f_j(K_j) = \emptyset$ for $i \ne j$, and14An *n*-dimensional oriented Riemannian manifold *M* naturally induces a current (on its geodesic metric space), that we will also denote by , given by integration of $\omega \in {\mathcal {D}}^{n}$ over *M*,15where $\tau$ is a (unit) orienting *n*-vector field. In this case, the mass of  equals the Riemannian volume.

The intrinsic representation of rectifiable currents by Ambrosio and Kirchheim [1, Theorem 9.1] shows that at least in some sense, this formula holds for any integer rectifiable current. More precisely, if *Z* is a $w^*$-separable dual space (i.e. $Z = G^*$ for a separable Banach space *G*), and *T* is an integer rectifiable current on *Z*, then there exists a countably ${{\mathcal {H}}}^n$-rectifiable set $Y\subset Z$, a Borel function $\theta _T: Y \rightarrow {\mathbb {N}}$ (which we call the multiplicity of *T*) with $\int _Y \theta _T \, d{{\mathcal {H}}}^n < \infty$ and an orientation $\tau$ of *Y* such that

16 $$\begin{aligned} T(f , \pi _1, \dots , \pi _n) = \int _Y f(z) \theta _T(z) \langle d\pi _1 \wedge \dots \wedge d\pi _n, \tau \rangle \, d{{\mathcal {H}}}^n(z), \end{aligned}$$

for $(f, \pi _1, \dots , \pi _n) \in {\mathcal {D}}^n(Z)$. We sometimes write . The multiplicity $\theta _T$ corresponds to the $\theta _i$ in the parametric representation (14), in the sense that for $\Vert T\Vert$-a.e. $x \in f_i(K_i)$, $\theta (x) = \theta _i$.

Moreover, the mass of *T* satisfies

17 $$\begin{aligned} \Vert T\Vert = \lambda \theta _T {{\mathcal {H}}}^n \llcorner Y, \end{aligned}$$

for a Borel function $\lambda : Y \rightarrow [c(n), C(n)]$ (the area factor) that is bounded away from zero and infinity by constants that only depend on the dimension.

It is true that we have not defined the objects appearing in (16). For a precise definition and formulation see [1]. For the purpose of the paper we just would like to stress the analogy with the formula for a current induced by a Riemannian manifold.

Additionally, the representation formula makes clear that if there is another integral current *S* supported on a subset of *Y*, it holds that $S = T \llcorner (b / \theta _T)$ for a Borel function $b:Y \rightarrow {\mathbb {Z}}$. We will denote the ratio $(b/ \theta _T)$ by $\Delta S / \Delta T$.

On *Y*, a version of the Lebesgue differentiation theorem is still valid. By [2, Theorem 5.4] and the remark following it, if $A \subset Y$ is Borel, then for ${{\mathcal {H}}}^n$-a.e. $x \notin A$,

18 $$\begin{aligned} \Theta _n( \Vert T \llcorner \, A \Vert , x ) = \Theta _n(\Vert T\Vert \llcorner A, x) = 0, \end{aligned}$$

while on the other hand for every Borel function $g:Y \rightarrow {\mathbb {R}}$, for ${{\mathcal {H}}}^n$-a.e. $x \in Y$,

19 $$\begin{aligned} \Theta _n(\Vert T \llcorner g\Vert , x) = \lambda (x) \theta _T(x) g(x). \end{aligned}$$

A very useful technique in dealing with currents on metric spaces is called slicing. For the purpose of this manuscript, we only need zero-dimensional slices. For $T \in I_n(X)$, a Lipschitz map $\Phi : X \rightarrow {\mathbb {R}}^n$ and points $x \in {\mathbb {R}}^n$, the slices $\langle T, \Phi , x \rangle \in I_0(X)$ are characterized by the property

20 $$\begin{aligned} \int _{{\mathbb {R}}^n} \langle T, \Phi , x \rangle \psi (x) \, dx = T \llcorner (\psi \circ \Phi ) \, d \Phi , \qquad \text {for all } \psi \in C_c\left( {\mathbb {R}}^n\right) , \end{aligned}$$

from which it follows that for any bounded Borel function *f* on *X*,

21 $$\begin{aligned} \int _{{\mathbb {R}}^n} \langle T, \Phi , x \rangle (f) \, dx = T (f d\Phi ). \end{aligned}$$

By [1, Theorem 9.7], if *X* is in addition a $w^*$-separable dual space, and , for a rectifiable set $Y \subset X$, the slices are very easy to interpret. Indeed, for ${{\mathcal {L}}}^n$-a.e. $x \in {\mathbb {R}}^n$, $\Phi ^{-1}(x)\cap Y$ contains at most finitely many points and

22 $$\begin{aligned} \langle T, \Phi , x \rangle = \sum _{p \in \Phi ^{-1}(x)\cap Y} a_p \theta _T(p) \delta _p, \end{aligned}$$

for some choice of $a_p \in \{ - 1, 1 \}$. Finally, for instance by [1, Theorem 5.7] if $S \in I_n(X)$, with ${{\mathcal {H}}}^n(\mathrm{{set}}(T) \backslash \mathrm{{set}}(S)) = 0$, for ${{\mathcal {L}}}^n$-a.e. $x \in {\mathbb {R}}^n$,

23 $$\begin{aligned} \langle S, \Phi , x \rangle = \langle T, \Phi , x \rangle \llcorner \frac{\Delta S}{\Delta T}. \end{aligned}$$

Finally, we note that a separable space *Y* can always be isometrically embedded into a $w^*$-separable Banach space. Indeed, if $y_i$ ($i=1, 2, \dots$) is a dense sequence in *Y*, we may embed *Y* into the $w^*$-separable Banach space $L^\infty (\{ y_i \}_i)$ by a Kuratowski embedding $I: Y \rightarrow L^\infty (\{y_i\}_i)$ given by

24 $$\begin{aligned} (I(y))(y_i) := d(y, y_i) - d(y, y_1). \end{aligned}$$

### Integral Current Spaces and Intrinsic Flat Convergence

Let $S, T \in I_n(X)$. The flat distance between *S* and *T* in *X* is defined as

25 $$\begin{aligned} d_F^X(S, T) := \inf \{ {{\mathbf {M}}}(U) + {{\mathbf {M}}}(V) \, | \, S - T = U + \partial V, \, U \in I_n(X), \, V \in I_{n+1}(X) \}.\nonumber \\ \end{aligned}$$

As briefly mentioned in the introduction, an *n*-dimensional integral current space $(X, d_X, T)$ is a pair of a metric space $(X, d_X)$, which is not necessarily complete, and a current $T \in I_n({\bar{X}})$ on the completion of *X*. Additionally, by convention, it is assumed that the current is completely settled, that is $X = \mathrm{{set}}(T)$.

The intrinsic flat distance between two integral current spaces $(X, d_X, T)$ and $(Y, d_Y, S)$ is given by26If $d_{\mathcal {F}}( (X, d_X, T), (Y, d_Y, S)) = 0$, this implies that there exists a current-preserving isometry $\phi : X \rightarrow Y$, that is an isometry such that $\phi _\# T = S$. Note that without the convention $X = \mathrm{{set}}(T)$ (or a similar condition), this would certainly not be the case in general.

We will denote the metric space of (equivalence classes of) *n*-dimensional integral current spaces with the intrinsic flat distance by ${\mathcal {M}}_n^{IF}$.

There is an involution $\iota$ acting on ${\mathcal {M}}^{IF}_n$, given by

27 $$\begin{aligned} \iota ( X, d_X, T) := (X, d_X, - T). \end{aligned}$$

We may endow the quotient space ${\mathcal {M}}^{IF}_n/\iota$ by the quotient distance:

28 $$\begin{aligned} \begin{aligned} d_{{\mathcal {F}}/\iota } (M, N) := \inf \{ d_{\mathcal {F}} (M, \iota ^{\epsilon _1} M_1)&+ d_{\mathcal {F}}(M_1, \iota ^{\epsilon _2} M_2) + \dots + d_{\mathcal {F}}(M_{n-1},\iota ^{\epsilon _n} N) \\&\, | \, M_i \in {\mathcal {M}}^{IF}_n, \epsilon _i \in \{0,1\} \} \end{aligned} \end{aligned}$$

In general, such a definition only yields a pseudometric. However, in this special case, the quotient distance is indeed a distance, and is in fact given by

29 $$\begin{aligned} d_{{\mathcal {F}}/\iota } (M_1, M_2 ) = \min ( d_{\mathcal {F}}(M_1, M_2) , d_{\mathcal {F}}(M_1, \iota (M_2)) ). \end{aligned}$$

We denote the metric space ${\mathcal {M}}_n^{IF}/\iota$ endowed with this quotient metric by ${\mathcal {M}}_n^{IF/\iota }$.

### The Structure of Spaces with Ricci Curvature Bounded Below

In [3–5], Cheeger and Colding study the structure of metric spaces that arise as the Gromov-Hausdorff limits of manifolds with Ricci curvature uniformly bounded from below.

Cheeger and Colding consider both noncollapsed and collapsed limit spaces. From the point of view of intrinsic flat convergence, the collapsed case is trivial as in that case the approximating sequence of Riemannian manifolds converges in the intrinsic flat distance to the zero integral current space. We therefore consider only noncollapsed limit spaces, for which the results by Cheeger and Colding are much stronger.

We borrow the following definitions. If $X \in {\mathcal {M}}^{GH}(n, \Lambda , v, D)$, and $p \in X$, we say $p \in {\mathcal {R}}_{\epsilon , \delta }$ if and only if

30 $$\begin{aligned} d_{GH}(B_r(p), B_r(0) )< \epsilon r, \quad \text {for all } r < \delta . \end{aligned}$$

We further define the regular set ${\mathcal {R}}$ by

31 $$\begin{aligned} {\mathcal {R}} := \bigcap _{\epsilon> 0} \bigcup _{\delta > 0} {\mathcal {R}}_{\epsilon , \delta }. \end{aligned}$$

In words, $p \in X$ is regular if for all $\epsilon > 0$ there exists a $\delta > 0$ such that $B_r(p)$ is $\epsilon$-close to a Euclidean ball at every scale smaller than $\delta$.

Cheeger and Colding show that in the noncollapsed case, the Hausdorff codimension of the complement $X \backslash {\mathcal {R}}$ is at least 2. They use this to prove in [4, Corollary 3.9 and 3.10] a (local) connectedness result. More precisely, for all $q_1, q_2 \in {\mathcal {R}}$, and every $\epsilon , \sigma > 0$, there is a $\delta > 0$ such that there is a path in ${\mathcal {R}}_{\epsilon , \delta }$ of length smaller than $d(q_1, q_2) + \sigma$ connecting $q_1$ and $q_2$. It follows immediately that when $q \in X$, $q_1, q_2 \in B_t(q) \cap {\mathcal {R}}$, for every $\epsilon > 0$ there is a path in ${\mathcal {R}}_{\epsilon , \delta }$ connecting $q_1$ and $q_2$ that remains inside $B_t(q)$. Indeed, there is a $\sigma > 0$ such that $q_1, q_2 \in B_{t- 3\sigma }(q)$. Next, there exists a $q_3 \in {\mathcal {R}}$ with $d(q_3, q) < \sigma$ by the Bishop-Gromov estimate and the fact that ${\mathcal {R}}$ has full measure. By the above, there exists a $\delta > 0$ such that there are paths in ${\mathcal {R}}_{\epsilon , \delta }$ from $q_1$ to $q_3$ and from $q_3$ to $q_2$ respectively, both of length smaller than $t - \sigma$, as for $k = 1, 2$,

32 $$\begin{aligned} d(q_k, q_3) \le d(q_k , q) + d(q, q_3) < t - 3 \sigma + \sigma = t - 2\sigma . \end{aligned}$$

These paths are contained in $B_t(q)$ and by concatenating them we obtain a path from $q_1$ to $q_2$ in $B_t(q) \cap {\mathcal {R}}_{\epsilon , \delta }$.

## Degree Estimate

For an integral current $T \in I_n(X)$ on a complete metric space *X* and a Lipschitz $\Psi : X \rightarrow {\mathbb {R}}^n$ the pushforward $\Psi _\# T \in I_n({\mathbb {R}}^n)$ is represented by a unique BV function, by a representation theorem due to Ambrosio and Kirchheim [1, Theorem 3.7]. The theorem also ensures that the mass measure of the boundary of $\Psi _\# T$ equals the total variation of the distributional derivative of the representing function. We call such a function *the degree* of $\Psi$ with respect to *T*.

### Definition 2.1

Let *X* be a complete metric space and let $T \in I_n(X)$. Let $\Psi :X \rightarrow {\mathbb {R}}^n$ be a Lipschitz map. We define the degree of $\Psi$ with respect to *T* as the (unique) function $\deg (T, \Psi , .) \in \mathrm {BV}({\mathbb {R}}^n)$, taking values in ${\mathbb {Z}}$, that satisfies


If *T* is a current induced by an oriented Riemannian manifold, then the degree defined above indeed corresponds to the usual topological degree.

By the Ambrosio-Kirchheim slicing theorem [1, Theorem 5.7] and [1, Eq. (5.18)] the degree can be evaluated by

33 $$\begin{aligned} \begin{aligned} \deg ( T, \Psi , x)&= \langle \Psi _\# T, \mathrm {Id}, x \rangle (1) \\&= \Psi _\# \langle T, \Psi , x \rangle (1) \\&= \langle T, \Psi , x \rangle (1). \end{aligned} \end{aligned}$$

This observation is very useful in light of the estimates by Sormani and the second author that show that integrals of flat distances between slices of two currents can be controlled by the flat distance between the full currents. This translates to an $L^1$ estimate on the difference between the degrees.

### Lemma 2.2

Let *Z* be a complete metric space and let $T^k \in I_n(Z)$, $k=1,2$. Let $\Phi ^k: Z \rightarrow {\mathbb {R}}^n$ be such that every component $\Phi ^k_j$ is 1-Lipschitz. Then

34 $$\begin{aligned} \begin{aligned} \Vert \deg \left( T^1, \Phi ^1, .\right)&- \deg \left( T^2, \Phi ^2, .\right) \Vert _{L^1\left( {\mathbb {R}}^n\right) } \le d_F\left( T^1 , T^2 \right) \\&+ 2 \sum _{j=1}^n \Vert \Phi ^1_j - \Phi ^2_j\Vert _\infty \left( {{\mathbf {M}}}\left( T^2\right) + {{\mathbf {M}}}\left( \partial T^2\right) \right) . \end{aligned} \end{aligned}$$

### Proof

We use the estimate by Sormani and the second author [10, Proposition 4.17]

35 $$\begin{aligned} \begin{aligned} \int _{{\mathbb {R}}^n}&d_F \left( \langle T^1, \Phi ^1, x \rangle , \langle T^2, \Phi ^2, x \rangle \right) \, dx \le d_F \left( T^1, T^2 \right) \\&\quad + 2 \sum _{j=1}^n \Vert \Phi ^1_j - \Phi ^2_j \Vert _\infty \left[ {{\mathbf {M}}}\left( T^2\right) + {{\mathbf {M}}}\left( \partial T^2\right) \right] . \end{aligned} \end{aligned}$$

From this inequality, we conclude the estimate (34) by using the link between slices and the degree explained in (33). Indeed, for ${{\mathcal {L}}}^n$-a.e. $x\in {\mathbb {R}}^n$,

36 $$\begin{aligned} \begin{aligned} \left| \deg \left( T^1, \Phi ^1, x\right) - \deg \left( T^2 , \Phi ^2, x\right) \right|&\le \left| \langle T^1, \Phi ^1, x \rangle \left( 1\right) - \langle T^2, \Phi ^2, x \rangle \left( 1\right) \right| \\&\le d_F\left( \langle T^1, \Phi ^1, x \rangle , \langle T^2, \Phi ^2, x \rangle \right) . \end{aligned} \end{aligned}$$

$$\square $$

## A Consequence of Colding’s Volume Estimate

Throughout this section we use the notation $B_r(0)$ to denote the open ball of radius *r* centered at 0 in the Euclidean space ${\mathbb {R}}^n$. Let *X* be a metric space, $x \in X$, and a radius $R > 0$, such that

37 $$\begin{aligned} d_{\mathrm {GH}} (B_R(x), B_R(0)) < \epsilon . \end{aligned}$$

Let $\{x_1, \dots , x_n\} \subset X$ and consider the map $\Phi _{x, x_1, \dots ,x_n}$ given by

38 $$\begin{aligned} \Phi _{x, x_1, \dots , x_n} (y) = \left( d_M( x_1, y) - d_M(x_1, x) ,\dots ,d_M(x_n, y)- d_M(x_n, x) \right) . \end{aligned}$$

We call $\Phi _{x, x_1, \dots , x_n}$ an $(\epsilon ,R)$-chart around *x* if there exist a metric space *Z* and isometric embeddings $\phi : B_R(x) \rightarrow Z$ and $\psi : B_R(0) \rightarrow Z$, such that

39 $$\begin{aligned} d^Z_H(\phi (B_R(x)), \psi (B_R(0))) < \epsilon , \end{aligned}$$

and

40 $$\begin{aligned} d_H^Z(\phi (\{x, x_1, \dots , x_n\}), \psi (\{0, Re_1, \dots , Re_n\})) < \epsilon , \end{aligned}$$

where $\{e_i\}$ is the standard orthonormal frame in ${\mathbb {R}}^n$, and $d_H^Z$ denotes the Hausdorff distance in *Z*.

Note that every coordinate function of $\Phi _{x, x_1, \dots , x_n}$ is Lipschitz with Lipschitz constant 1.

In [6, Sect. 2], Colding shows the following result, that we phrase as a lemma.

### Lemma 3.1

(Colding [6]). Let $\eta > 0$. There exist $\Lambda = \Lambda (\eta , n) > 0$, $R = R(\eta , n) > 1$, and $\epsilon = \epsilon (\eta , n) > 0$ with the following property. For every *n*-dimensional complete Riemannian manifold *M* with ${{\mathrm{Ric}}}_{M} \ge -(n-1) \Lambda$, with $p \in M$, such that $d_{\mathrm {GH}}(B_R(p), B_R(0) ) < \epsilon$, and every $(\epsilon ,R)$-chart $\Phi$ around *p*, it holds that

41 $$\begin{aligned} {{\mathcal {L}}}^n\left( B_1(0) \backslash \Phi (B_1(p))\right) < \eta ^n/3 \end{aligned}$$

and

42 $$\begin{aligned} {{\mathcal {L}}}^n(\Phi (B_1(p))) \le {{\mathcal {H}}}^n(B_1(p)) < \omega _n + \eta ^n/3. \end{aligned}$$

From the above lemma, we can derive that on a large set, the $(\epsilon , R)$-chart $\Phi$ is locally one-to-one.

### Lemma 3.2

Let $\eta > 0$. There exist $\Lambda = \Lambda (\eta , n)$, $R = R(\eta , n)$ and $\epsilon = \epsilon (\eta , n)$ with the following property. For every *n*-dimensional, oriented, complete Riemannian manifold *M* with ${{\mathrm{Ric}}}_M \ge -(n-1) \Lambda$, $p \in M$ such that

$$\begin{aligned} d_{\mathrm {GH}} (B_R(p), B_R(0)) < \epsilon , \end{aligned}$$

and every $(\epsilon ,R)$-chart $\Phi$ around *p*, the set

$$\begin{aligned} G = \left\{ x \in B_1(0) \, \big | \, {{\mathcal {H}}}^0(\Phi ^{-1}(x) \cap B_1(p) ) = 1\right\} \end{aligned}$$

satisfies

$$\begin{aligned} {{\mathcal {L}}}^n(G) > \omega _n - \eta ^n. \end{aligned}$$

### Proof

We choose the constants $\Lambda , R$ and $\epsilon$ as in Lemma 3.1, so that

43 $$\begin{aligned} \omega _n - \eta ^n/3< {{\mathcal {L}}}^n( \Phi (B_1(p) ) ) \le {{\mathcal {H}}}^n(B_1(p)) < \omega _n + \eta ^n/3. \end{aligned}$$

Since every component of the map $\Phi$ is 1-Lipschitz, the Jacobian $J\Phi$ of $\Phi$ is bounded, $|J \Phi | \le 1$. We apply the coarea formula and find

44 $$\begin{aligned} \begin{aligned} {{\mathcal {H}}}^n( B_1(p) )&\ge \int _{B_1(p)} |J \Phi | \, d{{\mathcal {H}}}^n \\&= \int _{{\mathbb {R}}^n} {{\mathcal {H}}}^0 \left( B_1(p) \cap \Phi ^{-1} (x) \right) dx \\&\ge {{\mathcal {L}}}^n(\Phi (B_1(p)) ). \end{aligned} \end{aligned}$$

Therefore,

45 $$\begin{aligned} {{\mathcal {L}}}^n \left( \left\{ x \in \Phi (B_1(p)) \, \big | \, {{\mathcal {H}}}^0( B_1(p)\cap \Phi ^{-1}(x) ) \ge 2 \right\} \right) < 2 \eta ^n /3 \end{aligned}$$

and consequently

46 $$\begin{aligned} {{\mathcal {L}}}^n \left( G \right) > \omega _n - \eta ^n. \end{aligned}$$

$$\square $$

### Lemma 3.3

Let $\eta >0$. There exist $\Lambda = \Lambda (n)$, $R = R(\eta ,n)$ and $\epsilon = \epsilon (\eta ,n)$ such that if *M* is an *n*-dimensional oriented complete Riemannian manifold with ${{\mathrm{Ric}}}_{M} \ge - (n-1)\Lambda$, $p \in M$, and

$$\begin{aligned} d_{\mathrm {GH}}( B_R(p), B_R(0) ) < \epsilon , \end{aligned}$$

then for every $(\epsilon , R)$-chart $\Phi$ around *p* and for every $x \in B_{1- \sigma }(0)$,

$$\begin{aligned} \deg (T \llcorner B_1(p), \Phi , x) = \deg (T \llcorner B_1(p), \Phi , 0) = \pm 1, \end{aligned}$$

where $\sigma = \sigma (\eta ,n)$ is defined by

$$\begin{aligned} \omega _n - {{\mathcal {L}}}^n(B_{1-\sigma }(0)) = \eta ^n. \end{aligned}$$

### Proof

We may without loss of generality assume that $\eta ^n \le \omega _n/4 =: {\bar{\eta }}^n$.

Note that $\Phi$ is a $\sigma$-Gromov-Hausdorff approximation, for *R* large enough and $\epsilon$ small enough, only depending on *n*.

We choose $R=R(\eta ,n)$ and $\epsilon = \epsilon (\eta , n)$ such that $\Phi$ is an $\sigma$-Gromov-Hausdorff approximation, and moreover

$$\begin{aligned} \Lambda _{3.3}(n) = \Lambda _{3.2}( {\bar{\eta }}, n), \qquad R_{3.3}(\eta , n) \ge R_{3.2}({\bar{\eta }}, n), \qquad \epsilon _{3.3}(\eta , n) \le \epsilon _{3.2}({\bar{\eta }}, n), \end{aligned}$$

where the subscripts indicated the lemma in which the constants are introduced.

Consequently, $\Phi (\partial B_1(p)) \cap B_{1-\sigma }(0) = \emptyset$ and $\deg (T\llcorner B_1(p), \Phi , .)$ is constant on $B_{1-\sigma }(0)$. By Lemma 3.2 and our choice of $\sigma$,

47 $$\begin{aligned} {{\mathcal {L}}}^n\left( \left\{ x \in B_{1-\sigma }(0) \,\big | \, {{\mathcal {H}}}^0(\Phi ^{-1}(x) \cap B_1(p) ) = 1 \right\} \right) > 0. \end{aligned}$$

Therefore,

48 $$\begin{aligned} |\deg (T \llcorner B_1(p), \Phi , 0)| = 1. \end{aligned}$$

$$\square $$

## Proof of the Main Theorems

Theorems A and B in the introduction are implied by the following theorem.

### Theorem 4.1

Let $M_i \in {\mathcal {M}}(n, K, v, D)$ ($i = 1, 2, \dots$) converge in the Gromov-Hausdorff distance to the (compact) metric space *X*. We assume without loss of generality that $M_i$ and *X* are isometrically embedded in a common $w^*$-separable Banach space *Z*.

Then,

(i) a subsequence of the associated integral current spaces $M_i$ converges in the flat distance to $(X, d_X , T)$, with $\mathrm{{set}}(T) = X$,
(ii) the mass $\Vert T\Vert$ equals ${{\mathcal {H}}}^n$, the Hausdorff measure on *X*,
(iii) *T* has multiplicity one ${{\mathcal {H}}}^n$-a.e.,
(iv) for every $q \in X$, every $t > 0$, and every $S \in I_n(X)$ with $\Vert \partial S\Vert (B_t(q)) = 0$, there exists an integer $k \in {\mathbb {Z}}$ such that $S = k T$ on $B_t(q)$.

Before we prove Theorem 4.1, we explain how it implies the theorems in the introduction. Certainly, Theorem B is a direct consequence of part (iv) in the above theorem.

The map $F:{\mathcal {M}}^{IF/\iota }(n, \Lambda , v, D) \rightarrow {\mathcal {M}}^{GH}(n, \Lambda , v, D)$ given by

$$\begin{aligned} (X, d_X, T) \rightarrow X \end{aligned}$$

considered in Theorem A is well-defined as Sormani and Wenger have shown that if the intrinsic flat distance between two integral current spaces is zero, there is a (current-preserving) isometry between the spaces [13]. Therefore, the map does not depend on the choice of representative. Moreover, by item (i) of Theorem 4.1, *X* is indeed a compact metric space.

The map *F* is one-to-one by (iv). A one-to-one continuous map from a compact to a Hausdorff space is automatically a homeomorphism. So the only non-trivial part of Theorem A left to show is the continuity of *F*.

If $(X_i, d_{X_i} , T_i) \in {\mathcal {M}}^{IF/\iota }(n, \Lambda , v, D)$ converge in the intrinsic flat distance to an integral current space $(X, d_X, T)$, there are Riemannian manifolds $M_i\in {\mathcal {M}}^{IF/\iota }(n, \Lambda , v, D)$ such that the associated integral current spaces  converge in the intrinsic flat distance to $(X, d_X, T)$ while49By the Gromov compactness and embedding theorems, we may isometrically embed  into a common metric space, in which a subsequence converges in the Hausdorff distance to a compact metric space *Y*. It suffices to show that *Y* is isometric to *X*.

Theorem 4.1 shows that  for some $S \in I_n(Y)$. Hence,

50 $$\begin{aligned} d_{\mathcal {F}}((X, d_X, T), (Y, d_Y, S)) = 0 \end{aligned}$$

and thus *Y* is isometric to *X*.

We will now prove Theorem 4.1.

### Proof

By the Ambrosio-Kirchheim compactness theorem [1, Theorem 5.2], a subsequence of the associated currents $T_i$ converges in the weak sense to an integral current *T* without boundary. A theorem by Wenger [14, Theorem 1.4] implies that the $T_i$ converge to *T* in the flat distance in *Z* as well.

Let $p \in X$ be a regular point, that is $p \in {\mathcal {R}} \subset X$. (The definition of the sets ${\mathcal {R}}$ and ${\mathcal {R}}_{\epsilon , \delta }$ are as in [4], see also Sect. 1.3). Let $\eta > 0$. Let $\Lambda (n)$, $\epsilon (\eta , n)$ and $R(\eta , n)$ be as in Lemma 3.3. Since $p \in {\mathcal {R}}$, there exists a number $0< \delta < \sqrt{ \Lambda / K }$ such that $p \in {\mathcal {R}}_{\epsilon / R, \delta R}$. In other words, for all $r < \delta$,

51 $$\begin{aligned} d_{GH}( B_{r R}(p), B_{r R}(0) ) < \epsilon r. \end{aligned}$$

We will now show that for a subsequence, we may localize the flat convergence to balls. Since $M_i \rightarrow X$ in the Hausdorff distance in *Z*, there exists a sequence $p_i \in M_i$ such that $p_i \rightarrow p$. By the proof of [11, Lemma 4.1], see also [10, Theorem 4.16], for yet another subsequence, for ${{\mathcal {L}}}^1$-a.e. radius $0< r < \delta$, the currents restricted to balls of radius *r* converge, that is

52 $$\begin{aligned} d_F ( T_i \llcorner B_r(p_i), T \llcorner B_r(p) ) \rightarrow 0. \end{aligned}$$

We choose $p^1, \dots , p^n \in X$ such that

53 $$\begin{aligned} \Phi = \Phi _{p, p^1, \dots , p^n} \end{aligned}$$

is an $(\epsilon r, r R)$-chart around *p*.

In order to prove (ii), we will lift this $(\epsilon , R)$-chart $\Phi$ to charts on the manifolds in the approximating sequence. By the results in the previous section, we have good control of the degree of these charts, and Lemma 2.2 allows us to pass this control to the limit. Estimates on the degree will immediately imply density estimates.

We argue as follows. Since $M_i \rightarrow X$ in the Hausdorff distance, there exist $p^j_i$, $j = 1, \dots , n$ with $p^j_i \rightarrow p^j$ as $i \rightarrow \infty$, such that $\Phi ^i := \Phi _{p^i, p^1_i, \dots , p^n_i}$ is an $(\epsilon ,R)$-chart around $p_i$ for *i* large enough. The maps $\Phi ^i$ converge to $\Phi$ uniformly by the triangle inequality.

By Lemma 2.2, $\deg ( T_i \llcorner B_r(p_i), \Phi ^i, .) \rightarrow \deg ( T \llcorner B_r(p),\Phi ,.)$ in $L^1({\mathbb {R}}^n)$ as $i \rightarrow \infty$. By this convergence result and Lemma 3.3, for all $x \in B_{(1-\sigma ) r}(0)$,

54 $$\begin{aligned} \deg (T \llcorner B_r(p), \Phi , x ) = \deg (T \llcorner B_r(p), \Phi , 0 ) = \pm 1. \end{aligned}$$

In particular,

55 $$\begin{aligned} \begin{aligned} \Vert T\Vert (B_r(p))&\ge \int _{B_{(1-\sigma )r}(0)} |\deg (T \llcorner B_r(p), \Phi , x)| \, dx \\&\ge \omega _n r^n - \eta ^n r^n. \end{aligned} \end{aligned}$$

Moreover, by lower-semicontinuity of the mass measure under weak convergence,

56 $$\begin{aligned} \Vert T\Vert (B_r(p)) \le \liminf _{i \rightarrow \infty } \Vert T_i\Vert (B_r(p_i)) \le \omega _n r^n + \eta ^n r^n /3. \end{aligned}$$

In particular, $p \in \mathrm{{set}}(T)$, and since $\eta >0$ was arbitrary, the density $\Theta _n(\Vert T\Vert ,p)$ of $\Vert T\Vert$ in *p* is 1. Since ${{\mathcal {H}}}^n(X \backslash {\mathcal {R}}) = 0$, in fact ${{\mathcal {H}}}^n = \Vert T\Vert$, which shows (ii).

By the results by Cheeger and Colding however, we know that ${{\mathcal {H}}}^n$ satisfies the Bishop-Gromov estimate, so that in particular, at *every* point in *X* the density of ${{\mathcal {H}}}^n (= \Vert T\Vert )$ is (strictly) positive. Consequently, $\mathrm{{set}}(T) = X$, which finishes the proof of (i).

Our next objective is to show (iii), namely that *T* has multiplicity one. To this end, we use that the measure of the set $\{|\deg (T\llcorner B_r(p), \Phi ,.)| =1\}$ is very close to $\Vert T\Vert (B_r(p))$. This can only happen if most points in the image of $\Phi$ have exactly one pre-image, where the multiplicity is one.

More precisely, since

57 $$\begin{aligned} \begin{aligned} (\omega _n - \eta ^n)r^n&= \int _{B_{(1-\sigma )r}(0)} |\deg (T \llcorner B_r(p) , \Phi , x) | \, dx \\&\le \int _{B_{(1-\sigma )r}(0)} {{\mathbf {M}}}( \langle T\llcorner B_r(p), \Phi , x \rangle ) \, dx \\&\le \Vert T\Vert (B_r(p)) \le (\omega _n + \eta ^n/3 )r^n, \end{aligned} \end{aligned}$$

if we define the “good” set $G \subset B_{(1-\sigma )r}(0)$ by

58 $$\begin{aligned} G := \left\{ x \in B_{(1-\sigma )r }(0) \, \big | \, {{\mathbf {M}}}(\langle T\llcorner B_r(p), \Phi , x \rangle ) = 1 \right\} , \end{aligned}$$

it follows that

59 $$\begin{aligned} {{\mathcal {L}}}^n( B_{(1-\sigma ) r} (0)\backslash G ) < 2 \eta ^n r^n. \end{aligned}$$

For ${{\mathcal {L}}}^n$-a.e. $x \in G$, there is a unique $p_x \in \mathrm{{set}}(T)\cap B_r(p)$ such that $\Phi (p_x) = x$ and the multiplicity $\theta _T(p_x) = 1$. Therefore, again using that $\Vert T\Vert (B_r(p)) \le \omega _n r^n+\eta ^n r^n /3$, we find

60 $$\begin{aligned} \Vert T\Vert ( \{ x \in B_r(p) \, | \, \theta _T(x) \ne 1 \} ) < 4 \eta ^n r^n. \end{aligned}$$

This shows that in every $p \in {\mathcal {R}}$, the density of the set where the multiplicity of *T* is larger than 1 equals zero. Since ${{\mathcal {H}}}^n( X \backslash {\mathcal {R}})= 0$, $\theta _T \equiv 1$. This shows (iii).

Finally, we need to show (iv). Let therefore $q \in X$ and $S \in I_n(X)$ such that $\Vert \partial S \Vert (B_t(q)) = 0$, for some $t > 0$. Let $p \in {\mathcal {R}} \cap B_t(q)$ and $r>0$ be as above, with the additional assumption that $r < d(p, X \backslash B_t(q))$. The representation theorem for integer rectifiable currents [1, Theorem 9.1] implies that there is a Borel function $\Delta S / \Delta T: X \rightarrow {\mathbb {Z}}$ such that

61 $$\begin{aligned} S = T \llcorner \frac{\Delta S}{\Delta T}. \end{aligned}$$

We will prove (iv) by showing that on a large set $\Delta S / \Delta T$ is equal to the ratio of two degrees, which in turn are constant. The next few lines will be devoted to the choice of a good representative for $\Delta S/ \Delta T$.

We first introduce an “average” version, based on a majority vote:

62 $$\begin{aligned} \left( \frac{\Delta S}{\Delta T}\right) _{p,r} := \arg \max _{k \in {\mathbb {Z}}} \Vert T\Vert \left( B_r(p) \cap \left\{ \frac{\Delta S}{\Delta T} = k \right\} \right) , \end{aligned}$$

where, if the maximum is not unique, we give preference to the smallest absolute value of *k*, and if this gives no decision, the positive value. However, this is quite irrelevant.

By the Lebesgue differentiation theorem, for ${{\mathcal {H}}}^n$-a.e. $q_1 \in X$,

63 $$\begin{aligned} \frac{\Delta S}{\Delta T} (q_1) := \lim _{r \rightarrow 0} \left( \frac{\Delta S}{\Delta T} \right) _{q_1,r}. \end{aligned}$$

For convenience, we will from now on only work with the precise representative of $\Delta S / \Delta T$ which we define to be the right-hand-side of (63) if this limit exists, and 0 otherwise.

By the characterization of slices [1, Theorems 5.7 and 9.7], for ${{\mathcal {L}}}^n$-a.e. $x \in G$, the slice $\langle T, \Phi , x \rangle$ is just a signed delta-measure,

64 $$\begin{aligned} \langle T \llcorner B_r(p), \Phi , x \rangle = \pm \delta _{p_x}, \end{aligned}$$

while

65 $$\begin{aligned} \langle S \llcorner B_r(p), \Phi , x \rangle = \frac{\Delta S}{\Delta T}(p_x) \langle T \llcorner B_r(p), \Phi , x \rangle . \end{aligned}$$

We apply both sides to the function identically equal to 1, and conclude that

66 $$\begin{aligned} \frac{\Delta S}{\Delta T} ( p_x ) = \frac{\deg (S \llcorner B_r(p), \Phi , x)}{ \deg (T \llcorner B_r(p), \Phi , x)}. \end{aligned}$$

If we inspect the proof of Lemma 3.3, we see that the constants $\Lambda , R$, and $\epsilon$ were chosen such that $\Phi$ is a $\sigma r$-Gromov-Hausdorff approximation. Hence, both $\deg (T \llcorner B_r(p), \Phi , .)$ and $\deg (S \llcorner B_r(p), \Phi , .)$ are constant on the ball $B_{(1-\sigma ) r}(0)$ and we can find a compact subset ${\tilde{G}} \subset G$, ${{\mathcal {L}}}^n({\tilde{G}}) > (\omega _n - 3 \eta ^n) r^n$ such that *every* $x \in {\tilde{G}}$ has exactly one pre-image $p_x$ under $\Phi$ in $\mathrm{{set}}(T) \cap B_r(p)$, and for this $p_x$

67 $$\begin{aligned} \frac{\Delta S}{\Delta T} ( p_x ) = \frac{\deg (S \llcorner B_r(p), \Phi , 0)}{ \deg (T \llcorner B_r(p), \Phi , 0)}. \end{aligned}$$

It follows that

68 $$\begin{aligned} \Vert T\Vert \left( \frac{ \Delta S}{\Delta T} \ne \left( \frac{\Delta S }{\Delta T}\right) _{p,r} \right)\le & {} \Vert T\Vert (B_r(p) \backslash \Phi ^{-1}({\tilde{G}})) \nonumber \\\le & {} \Vert T\Vert (B_r(p)) - \int _{{\tilde{G}}} |\deg (T \llcorner B_r(p), \Phi , x)| \, dx\\\le & {} \omega _n r^n + \eta ^n r^n / 3 - \omega _n r^n + 3 \eta ^n \nonumber \\\le & {} 4 \eta ^n r^n.\nonumber \end{aligned}$$

Therefore, for $\eta$ small enough, only depending on the dimension, the average $(\Delta S / \Delta T)_{p,r}$ is jointly continuous in (*p*, *r*) on the domain $p \in {\mathcal {R}}_{\epsilon / R, \delta R} \cap B_t(q)$, $0< r < \min (\delta , d_X(p, X \backslash B_t(q)) )$. In particular, $\Delta S/ \Delta T$ is continuous on ${\mathcal {R}}_{\epsilon / R, \delta R} \cap B_t(q)$.

However, by a result by Cheeger and Colding [4, Corollary 3.9 and 3.10] (see also Sect. 1.3), for all $q_1, q_2 \in B_t(q) \cap X$, there is a $\delta$ such that $q_1$ and $q_2$ lie in the same component of ${\mathcal {R}}_{\epsilon / R, \delta R} \cap B_t(q)$. Therefore, $\Delta S / \Delta T$ is constant on $B_t(p)$. This shows (iv). $\square$

### Remark 4.2

To obtain the necessary control on the degree, we use that an $(\epsilon , R)$-chart $\Phi$ is a Gromov-Hausdorff approximation. It is possible to conclude such control under weaker assumptions. A lot of information can be extracted from the existence of a map $\Psi : X \rightarrow {\mathbb {R}}^n$ on the limit space *X*, for which every component is 1-Lipschitz, and such that the excess $\Vert T\Vert (B_r(p)) - T(\chi _{B_r(p)} d\Psi )$ is small. We have decided to not present this argument in the manuscript, as it is considerably longer, and at this point, it is unclear whether there are applications in which the easier argument by a Gromov-Hausdorff approximation cannot be applied.
